# Rodents, not birds, dominate predation-related ecosystem services and disservices in vertebrate communities of agricultural landscapes

**DOI:** 10.1007/s00442-018-4242-z

**Published:** 2018-09-05

**Authors:** Matthias Tschumi, Johan Ekroos, Cecilia Hjort, Henrik G. Smith, Klaus Birkhofer

**Affiliations:** 1 Department of Biology, Lund University, Lund, Sweden; 2 Centre for Environmental and Climate Research, Lund University, Lund, Sweden; 3 Department of Ecology, Brandenburg University of Technology, Cottbus, Germany; 4 Present Address: Ecology Department, Swiss Ornithological Institute, Sempach, Switzerland

**Keywords:** Biological control, Predators, Semi-natural grasslands, Weed control, Wildlife cameras

## Abstract

To understand the relationship between conservation measures and agricultural yields, we need to know the contributions of organisms to both ecosystem services and disservices. We studied the activity and contribution of birds and mammals to intermediate ecosystem services (predation of weed seeds or invertebrate pests) and disservices (predation of crop seeds or beneficial invertebrates) in southern Sweden between June and November 2016. We measured seed and invertebrate predation rates using trays placed in front of 32 wildlife cameras in 16 cereal fields with a local habitat contrast (8 fields adjacent to another crop field and 8 fields adjacent to a semi-natural grassland) and along a landscape heterogeneity gradient (amount of semi-natural grassland). Both activity and predation were dominated by small mammals (mainly rodents), yet only a few species contributed to predation services and disservices according to camera records. Small mammal activity and predation varied considerably over time. Small mammal activity was significantly higher at trays with crop seeds or beneficial invertebrate prey compared to trays with pest prey, and crop seed predation by small mammals was significantly higher than weed seed predation. In contrast, bird activity and predation did not differ significantly between resource types, but varied over time depending on the habitat contrast. Predation of animal prey by birds was highest after cereal harvest, independent of habitat contrast. Our study highlights that birds and in particular rodents provide important intermediate ecosystem services, but also disservices, which fluctuate strongly in intensity over time.

**Electronic supplementary material:**

The online version of this article (10.1007/s00442-018-4242-z) contains supplementary material, which is available to authorized users.

## Introduction

Agricultural intensification and the resulting simplification of landscapes come at the cost of adverse effects on farmland biodiversity and changes in animal community composition (Kleijn et al. 2009; Gossner et al. 2016). These outcomes may negatively affect agricultural production, if communities become dominated by species that do not provide ecosystem services or even generate disservices (e.g. pests), rather than by species providing ecosystem services (e.g. natural enemies of pests; Sala et al. 2000; Sandbrook and Burgess 2015). Management options that aim at maximizing positive net effects between intermediate ecosystem services and disservices in agricultural landscapes thus need to identify service- and disservice-providing species in local communities and their habitat preferences and temporal dynamics.

Complex agricultural landscapes often harbour a high density and diversity of service-providing animals due to the availability of complementary resources (Macdonald et al. 2007; Kross et al. 2016). Mobile service-providing organisms rely on a combination of resources for foraging, reproduction or shelter from adverse conditions (Macdonald et al. 2007; Vickery and Arlettaz 2012; Smith et al. 2014). Different resource needs are rarely met in intensively managed crop fields alone and, as a consequence, many service providers also rely on semi-natural habitats such as hedges, flower strips or semi-natural grasslands (hereafter SNG; Pärt and Söderström 1999; Smith et al. 2014).

To preserve biodiversity and ecosystem services in farmland, agri-environment schemes often entail payments for the conservation and creation of semi-natural habitats (Herzog et al. 2017), and the promotion of SNG is a popular measure in many European countries (e.g. Sweden; Ekroos et al. 2014; Josefsson et al. 2017). While the effects of agri-environment schemes and general landscape complexity on biodiversity are relatively well known (Aviron et al. 2009; Kleijn et al. 2011), very little is known about their simultaneous effect on ecosystem services and disservices. In fact, fostering populations of service-providing species through habitat management may simultaneously affect the delivery of disservices (Gillespie and Wratten 2017). The limited knowledge about these relationships complicates predictions about net effects of management practices on intermediate ecosystem services.

Both birds and small mammals provide important intermediate services such as pest control, but some species of birds and mammals are also known to inflict severe damage to crops (Westerman et al. 2003a; Brown et al. 2007; Triplett et al. 2012; Whelan et al. 2015; Schäckermann et al. 2015; Şekercioğlu et al. 2016). For example, while many bird species are perceived as beneficial for pest control, corvids and rodents are primarily perceived as crop pests (Brown et al. 2007; Peisley et al. 2015). However, as corvids and rodents are facultative scavengers and consume weed seeds and animal prey, they may also contribute considerably to pest control (Elkinton et al. 2004; Whelan et al. 2008; Fischer and Schröder 2014; Young et al. 2014; Şekercioğlu et al. 2016). Studies focusing on predation services or disservices provided by vertebrates often consider the individual contribution of mammals or birds, but not their relative contributions (e.g. Westerman et al. 2003b; Baraibar et al. 2009). This limitation is further complicated by the fact that the involved species may switch diet during the season, resulting in variation of net effects by birds and mammals over time (Şekercioğlu et al. 2016). Understanding the net effects of bird and mammal species on predation-related intermediate ecosystem services and disservices across the growing season will help to improve conservation strategies that focus on the provision of intermediate ecosystem services.

To study the variation in ecosystem services and disservices over time, it may not suffice to assess the density or activity of functionally important species assemblages (Birkhofer et al. 2017). This is because densities of service providers not necessarily reflect ecosystem functions, for example because interactions between predator species affect predation services (Merfield et al. 2004; Weighill et al. 2017). Video surveillance is a promising method to quantify actual predation events and to assess the contribution of different species to predation services and disservices over time (Birkhofer et al. 2017).

This study aims to contribute to a better understanding of factors that affect predation services and disservices provided by birds and mammals throughout the crop growing season. The contribution of an implemented agri-environment measure on net predation services in agriculture was estimated in a field experiment in southern Sweden, using an underutilized approach to directly study predator activities. The following questions were addressed: (1) What is the relative contribution of birds and small mammals to predation services and disservices? (2) What are the dominant vertebrate predator species consuming crop seeds, weed seeds, beneficial prey and pest prey? (3) Is the activity and predation of birds and small mammals affected by local habitat configuration and landscape complexity? And (4) How do these effects vary over time?

## Materials and methods

### Study design

Field experiments were conducted in the province of Skåne, southern Sweden, in a region characterized by intensively farmed plains dominated by annually tilled crops and leys, interspersed with semi-natural habitats such as semi-natural grasslands. As part of our study design, we selected eight non-overlapping landscapes (1 km radius) along a landscape complexity gradient based on the cover of SNG prior to the experiments. Land-use information was obtained from the Swedish Board of Agriculture’s Integrated Administration and Control System database IACS. SNG were all un-improved grasslands receiving neither fertilizer nor pesticide inputs. In the study region, the occurrence of SNG was strongly associated with general landscape complexity (Persson et al. 2010). Within each landscape, we selected two spring-sown cereal fields (mean ± 1 SE area: 6.8 ± 1.0 ha), with one bordering a SNG (hereafter ‘SNG field’; mean ± 1 SE area of SNG: 3.9 ± 1.2 ha) and the other bordering another annual crop field (hereafter ‘control field’; design factor ‘habitat contrast’).

In each study field, we placed two flower pot trays (Hammarplast Botanica Ø 26 cm, border height 4 cm) and a motion-triggered wildlife camera (UOVision UV565HD, mounted on a wooden pole separated by 90 cm from the tray) in April 2016 in two plots separated by a distance of 40-70 m from each other along the intersection of the study field and the other bordering field (see Electronic Supplementary Material (ESM) Fig. S1 for a schematic illustration of the experimental setup). Trays and cameras were placed inside the cereal field (located 20 m away from the field border) in spring and early summer (i.e. pre-harvest—sample round 1 to 3; ESM Table S1; ESM Fig. S1) and moved to the field border in late summer and autumn (i.e. post-harvest—sample round 4 to 6; ESM Table S1; ESM Fig. S1). Moving the experimental setup was necessary as farmers managed fields after harvest and trays and cameras could not remain inside the crop fields. After harvest one SNG barley field had to be replaced with another nearby barley field bordering the same SNG. We drilled small holes (*n* = 80; Ø 1 mm) into each tray to allow for runoff of rain water. Nevertheless, heavy rains occasionally flooded some of the trays, but with no significant effect on the estimated activity or predation rates (ESM Table S2). Due to their curved border, flower pot trays successfully excluded the majority of ground-dwelling invertebrate predators such as ground beetles (MT, unpublished data). We additionally removed the vegetation bordering the trays at every visit to prevent vegetation to interfere with the cameras and to constraint invertebrate predators from climbing into the trays via the vegetation.

To assess predation rates by vertebrates, we used standardized plant and animal food resources placed in the trays (see Birkhofer et al. 2017). We used crop seeds (spring wheat—*Triticum aestivum* L. variety Diskett) and weed seeds (common hemp-nettle—*Galeopsis tetrahit* L. obtained from Herbiseed, Twyford, Berkshire, UK) to measure plant seed predation. As animal prey, we used potential beneficial prey (Earthworms—*Dendrobaena sp.*) and potential pest prey (Mealworms—*Tenebrio molitor* L.). Earthworms have long been recognized as ecosystem engineers beneficial to agricultural production due to their contribution to soil formation (Barley 1961). Mealworms, as the larvae of Tenebrionid beetles, are elateriform beetle larvae resembling wireworms (larvae of Elaterid beetles) which are among the most severe agricultural pests worldwide (Ritter and Richter 2013). Forty-nine plant seeds (either crop or weed seeds in rows of 7 × 7 seeds) were glued to each flower pot tray using double-sided sticky tape (TESA Carpet tape) and the remaining sticky tape was covered with a thin layer of sand. Animal prey (either five earthworms or five mealworms) was pinned alive to the trays. We attached the trays to the ground with three tent pegs.

In each sampling round (ESM Table S1), both plant resources were placed in all fields simultaneously. After seven days (eight days instead of seven in round five due to logistic constraints), we downloaded pictures recorded by the cameras to a portable computer and replaced seed trays by trays with either beneficial or pest prey. We collected data on animal resources after 2 days because of the limited survival of the prey after this period and, at the same time, we collected all recorded pictures. The experiment started in early June 2016, approximately 3 weeks after the cereal fields were sown. Three sample rounds were carried out before harvest (sample round 1 to 3; ESM Table S1) followed by three subsequent sample rounds after harvest, with the last round ending in early November (sample round 4 to 6; ESM Table S1).

Wildlife cameras recorded pictures of vertebrates at the trays during day and night (infrared flash). During each visit, the cameras recorded pictures at a constant rate as long as the vertebrate was present, often resulting in multiple pictures per visit. For each camera, sample round and resource type, we identified the visiting species (or taxonomic identity to the best possible resolution), the number of pictures recorded and the number of removed resource items per species. Comparing the pictures recorded before each visit of an individual to the pictures recorded after the visit allowed us to determine the number of removed prey items per species and visit.

### Statistical analyses

To avoid strongly zero-inflated models, we pooled species-specific data from all bird and all rodent species, respectively, to model overall bird and rodent activity and predation. Activity was defined as the total number of pictures showing visiting animals recorded by the camera. Predation was defined as the number of removed items identified from consecutive camera pictures depicting the same predator species. For both predator groups (rodents and birds), we fitted individual models for their activity at trays with the two (1) plant resources or (2) animal resources. Similar models were fitted for predation. For all models, we used permutational analysis of variance (PERMANOVA, Anderson 2001). All models included the fixed factors habitat contrast (2 levels), sample round (6 levels), resource type (2 levels) as well as the random blocking factor landscape ID and all two-way interactions. To specifically address the effect of pre- and post-harvest periods, we included an orthogonal contrast for pre (rounds 1–3) vs. post-harvest (rounds 4–6) sample rounds and the corresponding two-way interactions in all models. As patterns in rodent activity differed between individual resources (ESM Table S4), we additionally modelled individual plant (crop seeds/weed seeds) and animal (beneficial prey/pest prey) resources for rodents separately.

To assess the impact of landscape composition, we fitted separate models relating the SNG cover in a 1 km radius around each individual field to the field-averaged activity and predation of birds and rodents for each resource. We also included habitat contrast and the interaction of SNG cover and habitat contrast for modelling local vs. landscape interactions and landscape ID as random blocking factor. Due to the different duration that plant and animal resources were offered in fields and the fact that some cameras stopped recording because of technical failure or full memory cards, activity and predation were standardized for camera running time (expressed as activity or predation per hour that the camera was operational). All sessions with cameras running less than 24 h (*n* = 5) were excluded from the analyses. We used post hoc tests (pairwise PERMANOVA) to determine the significance between individual factor levels. Euclidean distances were used to calculate resemblance matrices and *P* values were obtained from 9999 permutations. All models were calculated using Primer-e version 7.0.12 and the PERMANOVA+ add-on (Clarke and Gorley 2015). PERMANOVA models were used as they do not rely on explicit assumptions about the distribution of the dependent variables (Anderson et al. 2008).

## Results

### Activity and predation rates: overall patterns

**Fig. 1:**
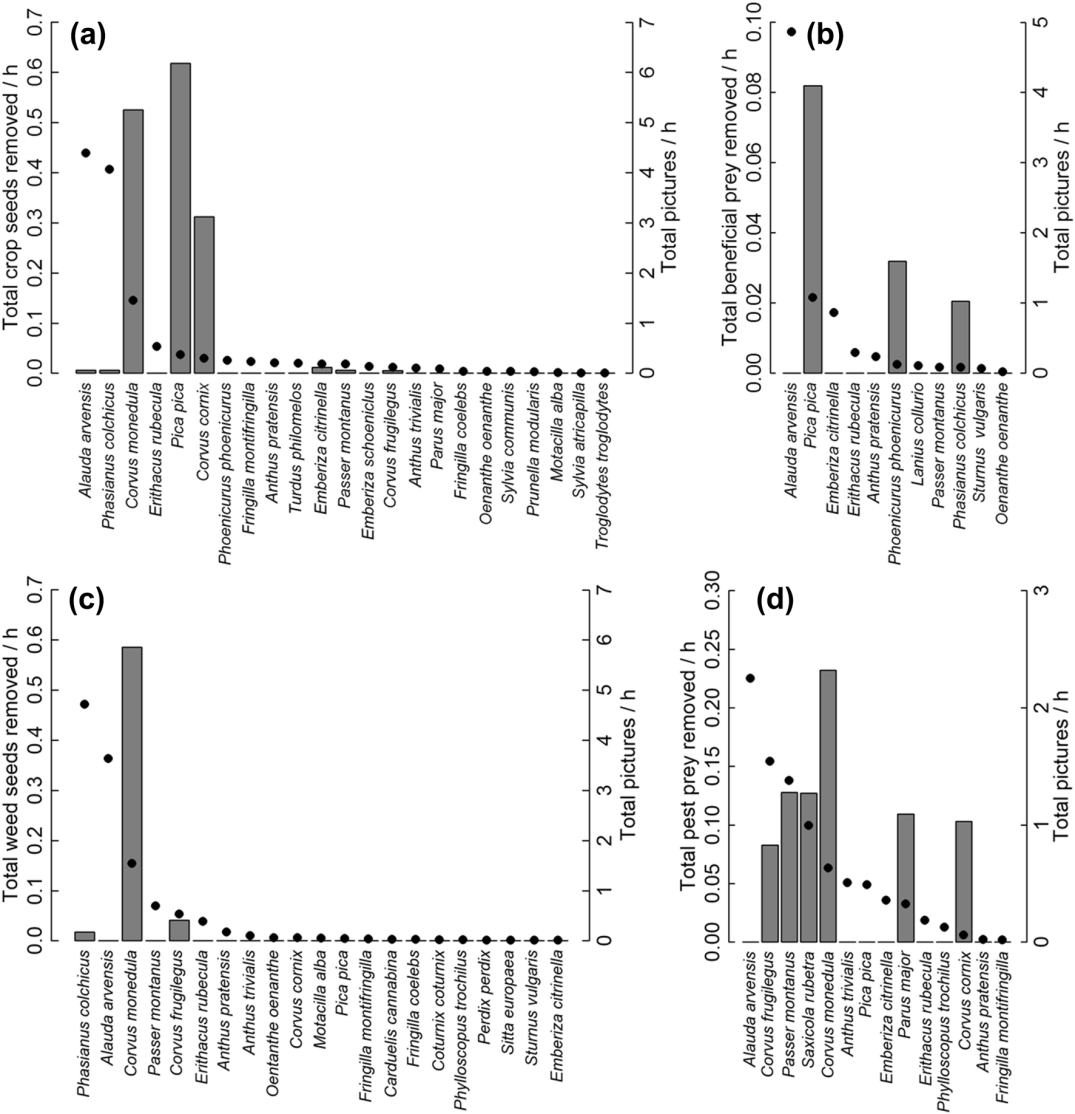
Predation rates by birds and bird activity as recorded by wildlife cameras. Total number of items removed per resource type (**a**–**d**) and hour by individual bird species (grey bars) and total number of pictures recorded per hour for the respective species (black dots) for **a** crop seeds, **b** beneficial prey, **c** weed seeds and **d** pest prey

We recorded a total of 31 bird species and 12 mammal taxa visiting the trays (Fig. 1; see ESM Table S3 for a list of all species). Of all recorded animal pictures (*n* = 43253), 88.1% (*n* = 38092) depicted mammals and 11.8% birds (*n* = 5092). A total of 67.1% of all provided crop seeds, 40.0% of weed seeds, 27.5% of beneficial prey and 38.0% of pest prey were predated. Of those, 89.9% were consumed by mammals and 10.1% by birds. With the exception of 17 pictures, all birds could be identified to species level. Mice, voles and shrews were not identified to species levels (summarized in the categories “mouse” and “shrew”, respectively). Voles could often not be differentiated from mice on camera pictures at night. In addition, there were 69 pictures showing animals (0.2%) that could not be identified at all.

Eleven bird species and three mammal taxa contributed to observed predation rates (Fig. 1). A total of 96.1% of the predation by birds was attributable to seven species: western jackdaws (*Corvus monedula*—38.4%), Eurasian magpies (*Pica pica*—18.5%), hooded crows (*Corvus cornix*—12.6%), tree sparrows (*Passer montanus*—7.4%), whinchats (*Saxicola rubetra*—7.3%), great tits (*Parus major*—6.1%) and rooks (*Corvus frugilegus*—5.7%). Predation by mammals was almost exclusively attributable to mice (94.8%) and brown rats (*Rattus norvegicus*—4.9%). The only other mammal that consumed resources was a single wild boar (*Sus scrofa*) that removed 16 crop seeds from a single tray. Bird activity and predation of individual species were not correlated on the community level (crop seeds: Pearson’s *r* = 0.065, *P *= 0.769, *n* = 23 species; weed seeds: *r* = 0.198, *P *= 0.391, *n* = 21 species; beneficial prey: *r* = − 0.004, *P *= 0.990, *n* = 11 species; pest prey: *r* = 0.189; *P *= 0.518, *n* = 14 species).

### Predictors of rodent activity

**Fig. 2:**
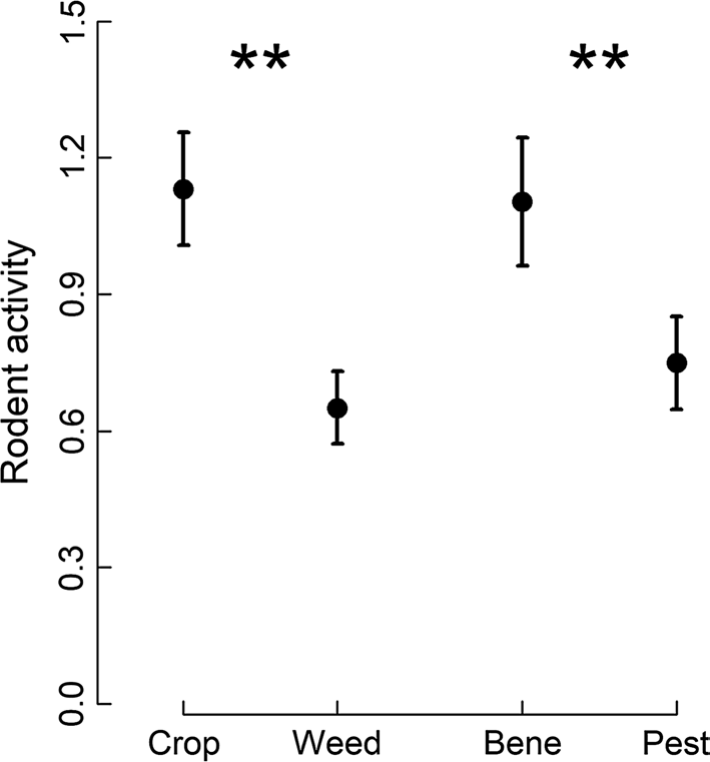
Rodent activity at different food resources. Mean (± 1 SE) standardized rodent activity (number of recorded pictures/h) at trays with crop seed (crop), weed seed (weed), beneficial prey (bene) and pest prey (pest) resources. Asterisks indicate a significant effect (*P* ≤ 0.01) between both seed resources or between both animal resources

**Fig. 3:**
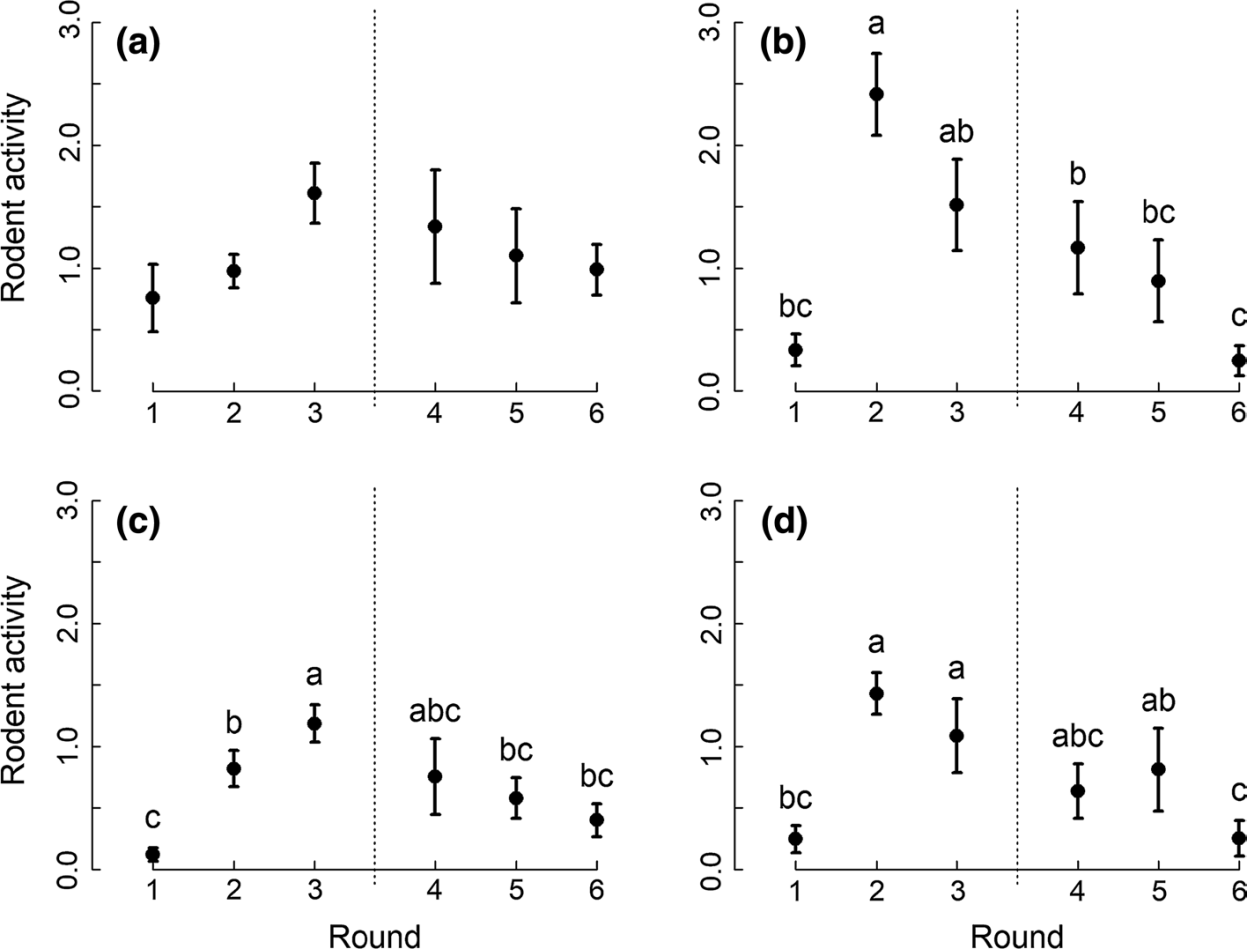
Rodent activity per sampling round at trays with different food resources. Mean (± 1 SE) standardized rodent activity (number of recorded pictures/h) at trays with **a** crop seeds, **b** beneficial prey, **c** weed seeds and **d** pest prey across the six rounds. Different letters indicate significant differences based on PERMANOVA post hoc tests (*P* ≤ 0.05) for resources with significant round effects. The vertical line represents the separation between pre- (rounds 1–3) and post-harvest (rounds 4–6)

Rodent activity at the trays differed significantly between resource types (ESM Table S4; Fig. 2). Rodent activity was significantly higher at trays with crop seeds than at trays containing weed seeds and significantly higher at trays with beneficial prey than at trays with pest prey (Fig. 2). When testing resources individually, rodent activity varied significantly between rounds at trays with weed seeds, beneficial prey and pest prey but not at trays with crop seeds (Table 1; Fig. 3). Rodent activity at trays with weed seeds increased steadily from the beginning of the season (early June; round 1) to harvest (Late July/Early August; round 3) and decreased gradually thereafter (Fig. 3c). A similar, yet non-significant pattern could be observed at trays with crop seeds (Fig. 3a). In contrast, rodent activity at trays with animal prey (beneficial and pest) increased markedly between early June (round 1) and early July (round 2) and decreased towards and after harvest (Fig. 3b,d). The decrease after harvest was more pronounced for trays with beneficial prey compared to pest prey and in both cases reached low levels in early November (round 6; Fig. 3b,d).

**Table 1:** Results of permutational analysis of variance (degrees of freedom *df*, pseudo-*F* and *P* values) for the effects of landscape ID, habitat contrast, round (including a pre-/post-harvest contrast) and two-way interactions on rodent activity at trays with different resource types (crop seeds; weed seeds; beneficial prey; pest prey)

	Crop seeds			Weed seeds			Beneficial prey			Pest prey		
	*df*	*F*	*P*	*df*	*F*	*P*	*df*	*F*	*P*	*df*	*F*	*P*
Landscape ID	*7*	*1.56*	*0.184*	*7*	*1.69*	*0.147*	*7*	*1.92*	*0.098*	*7*	*2.06*	*0.072*
Habitat contrast	1	0.07	0.789	1	0.05	0.833	1	0.09	0.767	1	1.16	0.318
Round	5	0.64	0.673	5	**4.68**	**0.003**	5	**6.67**	**< 0.001**	5	**4.55**	**0.002**
PRE/POST	1	0.00	0.985	1	0.82	0.389	1	5.53	0.054	1	2.98	0.126
Landscape ID × habitat contrast	*7*	*1.69*	*0.144*	*7*	***3.37***	***0.006***	*7*	*2.14*	*0.065*	*7*	*1.47*	*0.204*
Landscape ID × round	*35*	*1.58*	*0.099*	*35*	*0.88*	*0.657*	*35*	*1.31*	*0.214*	*35*	*1.00*	*0.494*
Landscape ID × PRE/POST	*7*	*1.59*	*0.149*	*7*	*1.40*	*0.224*	*7*	*1.03*	*0.424*	*7*	*1.25*	*0.283*
Habitat contrast × round	5	0.50	0.779	5	0.15	0.977	5	0.75	0.604	5	1.13	0.367
Habitat contrast × PRE/POST	1	0.49	0.495	1	0.16	0.693	1	1.27	0.271	1	0.38	0.534
Residual	34			33			34			34		
Total	94			93			94			94		

Models were fitted for different resources separately. Results of the global model assessing effects on plant and animal resources simultaneously are shown in ESM table S4

Significant effects (*P* ≤ 0.05) are shown in bold, random effects in italics, significant random effects (*P* ≤ 0.05) in bold italics and contrasts indented

### Predictors of bird activity

Bird activity did not differ significantly between resources (Table 2). However, there was a significant interaction of habitat contrast with round for both seeds and animal prey (Table 2; ESM Fig. S2). In addition, bird activity at trays with seed resources was differently affected by adjacent habitat type before compared to after harvest (Table 2).

**Table 2:** Results of permutational analysis of variance (degrees of freedom *df*, pseudo-*F* and *P* values) for the effects of landscape ID, habitat contrast, round (including a pre-/post-harvest contrast), resource and two-way interactions on bird activity at trays with seed and trays with animal prey resources

	Seed resources			Animal resources		
	*df*	*F*	*P*	*df*	*F*	*P*
Landscape ID	***7***	***3.62***	***0.002***	*7*	*1.07*	*0.395*
Habitat contrast	1	1.59	0.215	1	0.77	0.410
Round	5	1.04	0.401	5	0.88	0.540
PRE/POST	1	1.07	0.334	1	0.80	0.420
Resource	1	0.78	0.400	1	0.05	0.830
Landscape ID × habitat contrast	*7*	*4.37*	*<0.001*	*7*	*1.75*	*0.083*
Landscape ID × round	*35*	*2.75*	*<0.001*	*35*	*1.71*	**0.011**
Landscape ID × PRE/POST	*7*	*2.15*	*0.030*	*7*	*1.66*	*0.079*
Landscape ID × resource	*7*	*0.05*	*1.000*	*7*	*1.07*	*0.405*
Habitat contrast × round	5	**3.41**	**0.005**	**5**	**2.21**	**0.039**
Habitat contrast × PRE/POST	1	**5.78**	**0.012**	1	2.14	0.145
Habitat contrast × resource	1	0.17	0.686	1	0.06	0.830
Round × resource	5	0.17	0.973	5	0.61	0.734
PRE/POST × resource	1	0.01	0.911	1	0.85	0.408
Residual	114			115		
Total	188			189		

Models were fitted for seed and animal prey resources separately

Significant effects (*P* ≤ 0.05) are shown in bold, random effects in italics, significant random effects (*P* ≤ 0.05) in bold italics and contrasts indented

### Predictors of predation by rodents

**Fig. 4:**
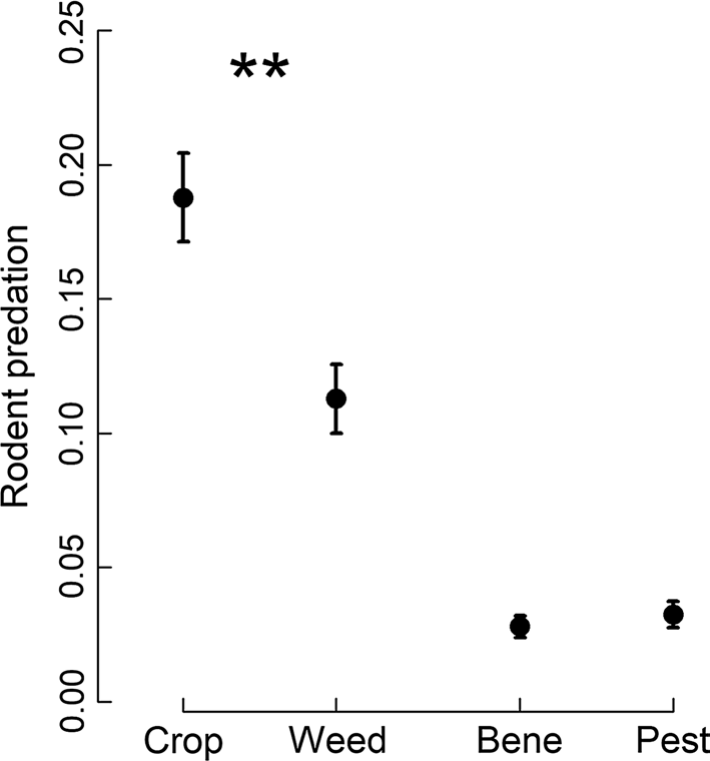
Predation of different food resources by rodents. Mean (± 1 SE) standardized predation (number of items removed/h) of crop seed (crop), weed seed (weed), beneficial prey (bene) and pest prey (pest) resources by rodents. Asterisks indicate a significant effect (*P* ≤ 0.01) between both seed resources

**Fig. 5:**
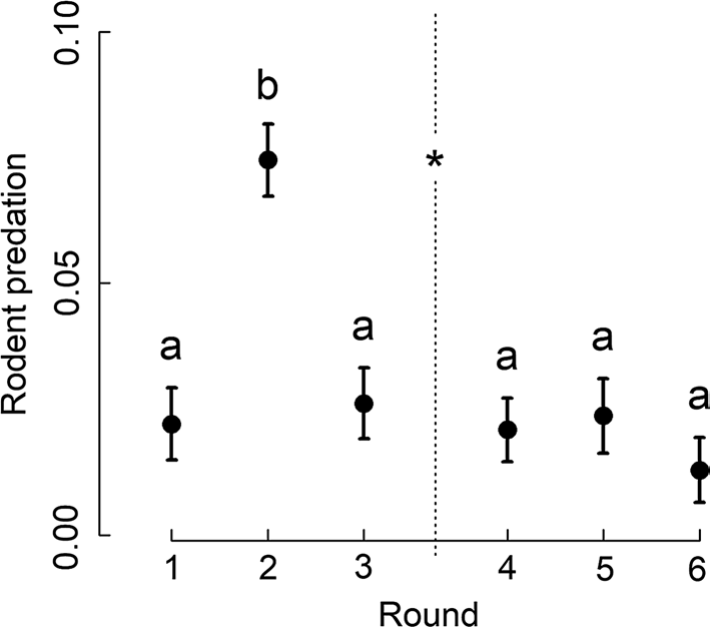
Predation of animal prey resources by rodents per sampling round. Mean (± 1 SE) standardized predation (number of items removed/h) of animal prey by rodents across the six rounds. Different letters indicate significant differences based on PERMANOVA post hoc tests (*P* ≤ 0.05). The vertical line represents the separation between pre- (rounds 1–3) and post-harvest (rounds 4–6) and the asterisk indicates a significant effect (*P* ≤ 0.05)

Predation by rodents was significantly higher for crop seeds compared to weed seeds, but did not differ significantly between animal prey resources (Table 3; Fig. 4). Since no other significant effects were found for seed resources, we did not analyse seed types individually. Predation of animal resources by rodents increased strongly between early June (round 1) and early July (round 2), followed by a decrease towards and after harvest (Fig. 5).

**Table 3:** Results of permutational analysis of variance (degrees of freedom *df*, pseudo-*F* and *P* values) for the effects of landscape ID, habitat contrast, round (including a pre-/post-harvest contrast), resource and two-way interactions on predation of seed and animal resources by rodents

	Seed resources			Animal resources		
	*df*	*F*	*P*	*df*	*F*	*P*
Landscape ID	*7*	*1.16*	*0.338*	*7*	***2.88***	***0.008***
Habitat contrast	1	3.10	0.126	1	0.15	0.703
Round	5	1.38	0.253	5	**8.41**	**< 0.001**
PRE/POST	1	1.91	0.198	1	**7.87**	**0.028**
Resource	1	**26.66**	**0.002**	1	0.63	0.460
Landscape ID × habitat contrast	*7*	***2.18***	***0.038***	*7*	*1.07*	*0.385*
Landscape ID × round	*35*	***2.42***	**< ** ***0.001***	*35*	*1.52*	*0.052*
Landscape ID × PRE/POST	*7*	*1.51*	*0.160*	*7*	*1.76*	*0.100*
Landscape ID × resource	*7*	*0.70*	*0.668*	*7*	*2.10*	*0.052*
Habitat contrast × round	5	1.58	0.173	5	1.02	0.407
Habitat contrast × PRE/POST	1	0.05	0.834	1	0.56	0.459
Habitat contrast × resource	1	0.25	0.625	1	0.69	0.408
Round × resource	5	1.53	0.188	5	1.43	0.220
PRE/POST × resource	1	0.14	0.712	1	0.32	0.583
Residual	114			115		
Total	188			189		

Models were fitted for seed and animal prey resources separately.

Significant effects (*P* ≤ 0.05) are shown in bold, random effects in italics, significant random effects (*P* ≤ 0.05) in bold italics and contrasts indented

### Predictors of predation by birds

Predation by birds did not differ significantly between resources (Table 4). However, predation of animal prey by birds was significantly higher after harvest (mean ± 1 SE: 0.0080 ± 0.0026 items removed/h) compared to before harvest (mean ± 1 SE: 0.0017 ± 0.0012 items removed/h; Table 4).

**Table 4:** Results of permutational analysis of variance (Degrees of freedom *df*, pseudo-*F* and *P* values) for the effects of landscape id, habitat contrast, round (including a pre-/post-harvest contrast), resource and two-way interactions on predation of seed and animal resources by birds

	Seed resources			Animal resources		
	*df*	*F*	*P*	*df*	*F*	*P*
Landscape ID	*7*	*1.90*	*0.072*	*7*	*0.89*	*0.515*
Habitat contrast	1	0.52	0.504	1	0.23	0.642
Round	5	1.47	0.198	5	1.37	0.249
PRE/POST	1	2.20	0.184	1	**7.81**	**0.028**
Resource	1	5.03	0.060	1	4.28	0.080
Landscape ID × habitat contrast	*7*	*0.87*	*0.538*	*7*	*1.09*	*0.377*
Landscape ID × round	*35*	***2.16***	***0.004***	*35*	*1.01*	*0.440*
Landscape ID × PRE/POST	*7*	***2.19***	***0.030***	*7*	*0.65*	*0.734*
Landscape ID × resource	*7*	*0.49*	*0.843*	*7*	*1.07*	*0.393*
Habitat contrast × round	5	1.12	0.357	5	0.69	0.633
Habitat contrast × PRE/POST	1	0.01	0.921	1	1.26	0.270
Habitat contrast × resource	1	0.43	0.516	1	0.08	0.782
Round × resource	5	0.26	0.935	5	0.70	0.621
PRE/POST × resource	1	0.07	0.794	1	2.46	0.121
Residual	114			115		
Total	188			189		

Models were fitted for plant and animal resources separately

Significant effects (*P* ≤ 0.05) are shown in bold, random effects in italics, significant random effects (*P* ≤ 0.05) in bold italics and contrasts indented

### Effects of landscape structure on rodents and birds

The amount of SNG in a 1 km radius did not affect the bird or rodent activity or predation results, neither directly nor by interactions with habitat contrast (ESM Table S5–S8). The effect of landscape structure on the predation of beneficial prey by birds could not be evaluated due to an insufficient number of observations for this resource.

## Discussion

Birds and rodents both contributed to predation services and disservices, but both activity at the trays and resource predation were strongly dominated by rodents. While the levels of bird activity were comparable between resources, activity of rodents was higher around trays with either of the two beneficial resources compared to activity around trays with potential pest resources. Accordingly, crop seed predation by rodents was higher than predation of weed seeds, but did not differ between invertebrate prey types. Rodent activity at trays with weed seeds and invertebrate prey, and predation of invertebrate prey by rodents, fluctuated significantly over the season. These findings highlight the importance of birds and in particular rodents for predation-related intermediate services and disservices.

The small contribution by birds in all experimental setups was surprising, given the relatively diverse bird communities at our study sites including large flocks of generalist birds feeding on seeds and invertebrates (MT unpublished data), and that a large body of literature has focussed on predation services provided by birds (Whelan et al. 2008; Mäntylä et al. 2011; Şekercioğlu et al. 2016). Due to the fact that rodents generally prefer dense vegetation (Tchabovsky et al. 2001; Fischer and Schröder 2014), but that most bird species avoid landing in dense crops, we expected birds to be more prominent in spring sown cereals compared to dense winter wheat fields (as in e.g. Westerman et al. 2003a; Baraibar et al. 2012; Fischer et al. 2017). Rodent densities in crop fields are often relatively low (Aschwanden et al. 2007; Fischer and Schröder 2014; Apolloni et al. 2017) and a previous study found low predation rates by rodents (e.g. Baraibar et al. 2012). The differences to our results may be explained by the timing of earlier predation studies (October in Baraibar et al. 2012) and the low density of suitable habitats for rodents in landscapes studied by Baraibar et al. (2012).

Although several studies have shown significant weed seed predation by rodents (Westerman et al. 2003a; Daedlow et al. 2013; Fischer et al. 2017), the general perception of rodents as crop pests is common in the scientific literature (Brown et al. 2007; Liu et al. 2012; Hauck et al. 2014). Compared to birds, rodents have received less attention as service providers. Indeed, the higher predation on crop compared to weed seeds in our study suggests a stronger contribution of rodents to disservices compared to pest control services. However, our results highlight that rodents are dominant contributors to both predation-related services and disservices, and positive effects may in the long-term outweigh disservices.

The net effects of seed and animal prey predation are context dependent and the resources used in this study and the experimental setup have obvious limitations. For example, the effect of weed seed predation will depend on weed species (i.e. some weeds can benefit biological control via positive effects on predators; Diehl et al. 2012) and farming system. Likewise, the predation of wheat seeds may allow conclusions about potential crop damage in cereal fields (Brown et al. 2003), but these effects likely vary between crops. In addition, net effects depend on the time of predation as for example post-harvest predation on crop seeds of the harvested crop could represent an ecosystem service rather than a disservice.

In terms of the applied methods, our results demonstrate that the predation of resources in arable fields cannot easily be predicted by activity as monitored by wildlife cameras. While predation of plant seeds caused by rodents was related to rodent activity, predation caused by individual bird species did not correlate with their activity around the trays. In general, field experiments on predation combined with camera monitoring may be among the few reliable methods to identify the contribution of individual functional groups to predation (Brown et al. 2016; Weighill et al. 2017; Birkhofer et al. 2017).

Only a small subset of the recorded species (ESM Table S3) actually contributed to predation services or disservices. This result is supported by previous studies that also identified a small number of vertebrate predators to drive the provision of services and disservices (Mols and Visser 2002; Peisley et al. 2015; Maas et al. 2015). In our study system, these species were mainly generalists known to utilize crop fields, with the majority of bird predators being corvid species. Although some taxa were observed to feed exclusively on pest resources (e.g. rooks, great tits and whinchats) or beneficial resources (e.g. Eurasian magpies) in our study, several species fed on both resource types (Fig. 1). This was also the case regarding the three most important predators: mice, rats and jackdaws (Fig. 1). Identifying the net effect of these key predators is important to plan strategies that aim for ecological intensification.

Previous studies have shown positive effects of landscape heterogeneity and semi-natural habitats on the abundance and richness of vertebrates and related services (Kross et al. 2016; Boesing et al. 2017). In our study, bird and rodent activity or predation were not significantly affected by habitat contrast or landscape complexity in most cases. Functional spillover of rodents and birds from SNG to crops may thus be limited in this context, or alternatively major predator species are generalists that do not strongly depend on semi-natural habitats (Aschwanden et al. 2007; Fischer and Schröder 2014; Garfinkel and Johnson 2015). Similar results suggesting limited spillover between SNG and crop fields and lack of positive effects on pest predation in adjacent cereal fields were also recently reported for invertebrate predators in southern Sweden (Birkhofer et al. 2018).

Rodent activity at trays with weed seeds and invertebrate prey, and predation of invertebrate prey by rodents varied over time. However, there was no temporal variability in seed predation by rodents or general resource predation by birds. The only exception for birds was higher predation of invertebrate prey after harvest. The pronounced increase of rodent activity from May to July corresponds with previously observed patterns for rodent population sizes in cereal fields (Tew and Macdonald 1993; Aschwanden et al. 2007). Rodents avoid habitats with low vegetation due to insufficient protection from predators and are thus rather found in semi-natural habitats compared to crop fields in early spring (Tchabovsky et al. 2001; Aschwanden et al. 2007; Fischer and Schröder 2014). Towards summer, rodents move into crop fields as they offer rich food sources and increasing protection from predators (Tew and Macdonald 1993; Aschwanden et al. 2007). During and after harvest, crop fields again offer reduced shelter (Tew and Macdonald 1993; Aschwanden et al. 2007).

High predation of animal prey by birds after harvest is unlikely a result of birds switching to an invertebrate diet in autumn, as the contrary should be expected for many bird species after breeding (e.g. Holland et al. 2006). Instead changing food availability or accessibility in the study fields during and after harvest could explain these differences (Best et al. 1990; Holmes and Froud-Williams 2005). In addition, the necessary relocating of trays to the field border could also have contributed to this result.

## Conclusions

The joint contribution of birds and rodents to predation-related services and disservices has so far rarely been quantified and no experimental field study has hitherto considered both plant and animal prey. Our results show that birds and mammals provide important pest control services in agricultural landscapes, and that rodents are particularly important in this context. Yet, birds and mammals may also contribute to disservices due to the predation of crop seeds and potentially beneficial invertebrates. Nevertheless, only few of the recorded taxonomic groups contributed substantially to predation services or disservices. In addition, we show that service and disservice levels provided by birds and mammals were unaffected by the studied local habitat contrast and landscape complexity. We conclude that the relative importance of small mammals has been underestimated in the past and that small mammals deserve more attention in agricultural measures aimed at supporting conservation biological control. The fact that rodents provided high levels of intermediate services and disservices over the whole study period and in all landscape settings questions their general perception as crop pests.

## Electronic supplementary material

Below is the link to the electronic supplementary material.

Supplementary material 1 (DOC 364 kb)
