# Supplementary material for: Rodents, not birds, dominate predation-related ecosystem services and disservices in vertebrate communities of agricultural landscapes
Source: Oecologia. 2018 Sep 5;188(3):863–73. doi: 10.1007/s00442-018-4242-z (PMC6208704; doi:10.1007/s00442-018-4242-z)
Supplement: Supplementary file 1 — Supplementary material 1 (DOC 364 kb) [file 442_2018_4242_MOESM1_ESM.doc]

## Electronic Supplementary Material

**Table S1.** Date, round number, timing in respect to crop harvest and location of the treatments within fields for the six experimental rounds.

| **Date** | **Round no.** | **Harvest** | **Location** |
| --- | --- | --- | --- |
| Early June | 1 | Before | Inside field |
| Early July | 2 | Before | Inside field |
| Late July / Early August | 3 | Before^1^ | Inside field^3^ |
| Late August | 4 | After^2^ | Field margin |
| Late September / Early October | 5 | After | Field margin |
| Early November | 6 | After | Field margin |

^1^two fields were already harvested during round no. four; ^2^six fields were harvested during or after round no. five; ^3^two fields (in the same landscape) had to be moved to the field margin for the last four days of round no. four.

**Table S2. Results of *t* tests for potential bias due to occasional flooding of trays. Shown are degrees of freedom *df*, *t*-values and *P*-values of Welch two sample *t* tests comparing activity and predation of trays that were eventually flooded to trays not flooded during the same round. Tests were performed for the rounds and resources where more than one tray was flooded.**

|  | *df* | *t* | *P* |
| --- | --- | --- | --- |
| **Activity of birds** |  |  |  |
| Crop seeds round 3 | 8.00 | -1.03 | 0.333 |
| Crop seeds round 4 | 8.09 | -1.29 | 0.233 |
| Weed seeds round 3 | 4.00 | 0.97 | 0.388 |
| Weed seeds round 4 | 10.01 | 1.03 | 0.328 |
| Beneficial prey round 4 | 10.29 | -1.23 | 0.244 |
| Pest prey round 4 | 11.36 | 0.57 | 0.580 |
|  |  |  |  |
| **Activity of rodents** |  |  |  |
| Crop seeds round 3 | 11.92 | 0.04 | 0.969 |
| Crop seeds round 4 | 13.76 | -2.05 | 0.060 |
| Weed seeds round 3 | 10.90 | -1.56 | 0.147 |
| Weed seeds round 4 | 5.45 | 0.17 | 0.871 |
| Beneficial prey round 4 | 10.67 | -1.41 | 0.187 |
| Pest prey round 4 | 13.15 | -0.07 | 0.942 |
|  |  |  |  |
| **Predation by birds** |  |  |  |
| ^1^Crop seeds round 3 | NA | NA | NA |
| Crop seeds round 4 | 6.00 | 1.33 | 0.231 |
| ^1^Weed seeds round 3 | NA | NA | NA |
| Weed seeds round 4 | 10.00 | 1.00 | 0.341 |
| Beneficial prey round 4 | 10.00 | -1.00 | 0.341 |
| Pest prey round 4 | 9.00 | 1.20 | 0.260 |
|  |  |  |  |
| **Predation by rodents** |  |  |  |
| Crop seeds round 3 | 12.88 | 0.40 | 0.697 |
| Crop seeds round 4 | 11.84 | -1.71 | 0.113 |
| Weed seeds round 3 | 7.33 | -0.88 | 0.408 |
| Weed seeds round 4 | 5.26 | 0.54 | 0.609 |
| Beneficial prey round 4 | 9.13 | -0.44 | 0.672 |
| Pest prey round 4 | 11.78 | 0.26 | 0.802 |

^1^Tests could not be performed as there was no predation by birds in these rounds

**Table S3.** Descriptive statistics of recorded camera data. Shown are total sums (Sum), as well as minima (Min), maxima (Max), arithmetic means (Mean) and standard deviations (SD) of camera recording periods (per camera and round) for the recorded number of pictures and the number of predated items (standardized by placed item number) per species or taxonomic group.

|  | **# OF PICTURES** | | | | |  | **# OF PREDATED ITEMS** | | | | |
| --- | --- | --- | --- | --- | --- | --- | --- | --- | --- | --- | --- |
|  | **Sum** | **Min** | **Max** | **Mean** | **SD** |  | **Sum** | **Min** | **Max** | **Mean** | **SD** |
| **Birds** |  |  |  |  |  |  |  |  |  |  |  |
| *Alauda arvensis* | 1685 | 0 | 577 | 4.39 | 40.86 |  | 0.02 | 0.00 | 0.02 | 0.00 | 0.00 |
| *Anthus pratensis* | 83 | 0 | 15 | 0.22 | 1.47 |  | 0.00 | 0.00 | 0.00 | 0.00 | 0.00 |
| *Anthus trivialis* | 56 | 0 | 24 | 0.15 | 1.48 |  | 0.00 | 0.00 | 0.00 | 0.00 | 0.00 |
| *Carduelis cannabina* | 5 | 0 | 5 | 0.01 | 0.25 |  | 0.00 | 0.00 | 0.00 | 0.00 | 0.00 |
| *Corvus cornix* | 62 | 0 | 35 | 0.16 | 1.92 |  | 2.08 | 0.00 | 1.00 | 0.01 | 0.07 |
| *Corvus frugilegus* | 175 | 0 | 53 | 0.46 | 3.67 |  | 0.94 | 0.00 | 0.80 | 0.00 | 0.04 |
| *Corvus monedula* | 550 | 0 | 97 | 1.43 | 8.65 |  | 6.32 | 0.00 | 1.00 | 0.02 | 0.11 |
| *Coturnix coturnix* | 3 | 0 | 3 | 0.01 | 0.15 |  | 0.00 | 0.00 | 0.00 | 0.00 | 0.00 |
| *Emberiza citrinella* | 91 | 0 | 24 | 0.24 | 1.85 |  | 0.04 | 0.00 | 0.04 | 0.00 | 0.00 |
| *Emberiza schoeniclus* | 22 | 0 | 22 | 0.06 | 1.12 |  | 0.00 | 0.00 | 0.00 | 0.00 | 0.00 |
| *Erithacus rubecula* | 199 | 0 | 63 | 0.52 | 4.57 |  | 0.00 | 0.00 | 0.00 | 0.00 | 0.00 |
| *Fringilla coelebs* | 12 | 0 | 5 | 0.03 | 0.36 |  | 0.00 | 0.00 | 0.00 | 0.00 | 0.00 |
| *Fringilla montifringilla* | 48 | 0 | 27 | 0.13 | 1.56 |  | 0.00 | 0.00 | 0.00 | 0.00 | 0.00 |
| *Lanius collurio* | 5 | 0 | 5 | 0.01 | 0.25 |  | 0.00 | 0.00 | 0.00 | 0.00 | 0.00 |
| *Motacilla alba* | 13 | 0 | 10 | 0.03 | 0.53 |  | 0.00 | 0.00 | 0.00 | 0.00 | 0.00 |
| *Oenanthe oenanthe* | 17 | 0 | 7 | 0.04 | 0.50 |  | 0.00 | 0.00 | 0.00 | 0.00 | 0.00 |
| *Parus major* | 30 | 0 | 15 | 0.08 | 0.99 |  | 1.00 | 0.00 | 1.00 | 0.00 | 0.05 |
| *Passer montanus* | 217 | 0 | 60 | 0.57 | 4.03 |  | 1.22 | 0.00 | 1.00 | 0.00 | 0.05 |
| *Perdix perdix* | 3 | 0 | 3 | 0.01 | 0.15 |  | 0.00 | 0.00 | 0.00 | 0.00 | 0.00 |
| *Phasianus colchicus* | 1484 | 0 | 777 | 3.86 | 52.09 |  | 0.28 | 0.00 | 0.20 | 0.00 | 0.01 |
| *Phoenicurus phoenicurus* | 49 | 0 | 43 | 0.13 | 2.21 |  | 0.30 | 0.00 | 0.30 | 0.00 | 0.02 |
| *Phylloscopus trochilus* | 9 | 0 | 6 | 0.02 | 0.34 |  | 0.00 | 0.00 | 0.00 | 0.00 | 0.00 |
| *Pica pica* | 150 | 0 | 38 | 0.39 | 2.92 |  | 3.05 | 0.00 | 1.00 | 0.01 | 0.08 |
| *Prunella modularis* | 6 | 0 | 6 | 0.02 | 0.31 |  | 0.00 | 0.00 | 0.00 | 0.00 | 0.00 |
| *Saxicola rubetra* | 47 | 0 | 38 | 0.12 | 1.99 |  | 1.20 | 0.00 | 1.00 | 0.00 | 0.05 |
| *Sitta europaea* | 3 | 0 | 3 | 0.01 | 0.15 |  | 0.00 | 0.00 | 0.00 | 0.00 | 0.00 |
| *Sturnus vulgaris* | 5 | 0 | 3 | 0.01 | 0.18 |  | 0.00 | 0.00 | 0.00 | 0.00 | 0.00 |
| *Sylvia atricapilla* | 1 | 0 | 1 | 0.00 | 0.05 |  | 0.00 | 0.00 | 0.00 | 0.00 | 0.00 |
| *Sylvia communis* | 6 | 0 | 3 | 0.02 | 0.22 |  | 0.00 | 0.00 | 0.00 | 0.00 | 0.00 |
| *Troglodytes troglodytes* | 1 | 0 | 1 | 0.00 | 0.05 |  | 0.00 | 0.00 | 0.00 | 0.00 | 0.00 |
| *Turdus philomelos* | 38 | 0 | 27 | 0.10 | 1.48 |  | 0.00 | 0.00 | 0.00 | 0.00 | 0.00 |
| Unknown bird | 17 | 0 | 6 | 0.04 | 0.43 |  | 0.00 | 0.00 | 0.00 | 0.00 | 0.00 |
| **Birds total** | **5092** | **0** | **777** | **13.26** | **67.31** |  | **16.46** | **0.00** | **1.00** | **0.04** | **0.18** |
|  |  |  |  |  |  |  |  |  |  |  |  |
| **Mammals** |  |  |  |  |  |  |  |  |  |  |  |
| *Canis lupus familiaris* | 4 | 0 | 2 | 0.01 | 0.12 |  | 0.00 | 0.00 | 0.00 | 0.00 | 0.00 |
| *Capreolus capreolus* | 52 | 0 | 13 | 0.14 | 1.07 |  | 0.00 | 0.00 | 0.00 | 0.00 | 0.00 |
| *Erinaceus europaeus* | 5 | 0 | 5 | 0.01 | 0.25 |  | 0.00 | 0.00 | 0.00 | 0.00 | 0.00 |
| *Felis silvestris catus* | 1592 | 0 | 156 | 4.15 | 14.47 |  | 0.00 | 0.00 | 0.00 | 0.00 | 0.00 |
| *Lepus europaeus* | 195 | 0 | 48 | 0.51 | 3.26 |  | 0.00 | 0.00 | 0.00 | 0.00 | 0.00 |
| *Meles meles* | 89 | 0 | 16 | 0.23 | 1.24 |  | 0.00 | 0.00 | 0.00 | 0.00 | 0.00 |
| Mouse | 34552 | 0 | 1117 | 89.98 | 135.01 |  | 139.49 | 0.00 | 1.10 | 0.36 | 0.43 |
| *Mustela putorius* | 17 | 0 | 15 | 0.04 | 0.77 |  | 0.00 | 0.00 | 0.00 | 0.00 | 0.00 |
| *Rattus norvegicus* | 1473 | 0 | 240 | 3.84 | 19.57 |  | 7.28 | 0.00 | 1.00 | 0.02 | 0.12 |
| Shrew | 60 | 0 | 13 | 0.16 | 0.89 |  | 0.00 | 0.00 | 0.00 | 0.00 | 0.00 |
| *Sus scrofa* | 16 | 0 | 6 | 0.04 | 0.41 |  | 0.41 | 0.00 | 0.41 | 0.00 | 0.02 |
| *Vulpes vulpes* | 37 | 0 | 24 | 0.10 | 1.27 |  | 0.00 | 0.00 | 0.00 | 0.00 | 0.00 |
| **Mammals total** | **38092** | **0** | **1291** | **99.20** | **142.80** |  | **147.18** | **0.00** | **1.10** | **0.38** | **0.44** |
|  |  |  |  |  |  |  |  |  |  |  |  |
| Unknown | 69 | 0 | 4 | 0.18 | 0.55 |  | 0.00 | 0.00 | 0.00 | 0.00 | 0.00 |
|  |  |  |  |  |  |  |  |  |  |  |  |
| **Total** | **43253** | **0** | **1314** | **112.66** | **165.26** |  | **163.64** | **0.00** | **1.10** | **0.43** | **0.44** |

**Table S4.** Results of permutational analysis of variance (Degrees of freedom *df*, pseudo-*F* and *P*-values) for the effects of landscape ID, habitat contrast, round (including a pre-/post-harvest contrast), resource and two-way interactions on rodent activity at trays with seed and animal resources. Significant effects (*P* ≤ 0.05) are shown in bold, random effects in italics and contrasts indented.

|  | **Seed resources** | | | **Animal resources** | | |
| --- | --- | --- | --- | --- | --- | --- |
|  | *df* | *F* | *P* | *df* | *F* | *P* |
| *Landscape ID* | *7* | ***2.24*** | ***0.036*** | *7* | ***3.66*** | ***0.001*** |
| Habitat contrast | 1 | 0.05 | 0.820 | 1 | 0.12 | 0.735 |
| Round | 5 | **2.58** | **0.046** | 5 | **9.73** | **<0.001** |
| PRE/POST | 1 | 0.10 | 0.757 | 1 | **6.72** | **0.037** |
| Resource | 1 | **18.14** | **0.006** | 1 | **15.63** | **0.007** |
| *Landscape ID x Habitat contrast* | *7* | ***3.64*** | ***0.001*** | *7* | ***2.83*** | ***0.010*** |
| *Landscape ID x Round* | *35* | ***1.57*** | ***0.040*** | *35* | *1.36* | *0.117* |
| *Landscape ID x PRE/POST* | *7* | *1.95* | *0.061* | *7* | *1.41* | *0.203* |
| *Landscape ID x Resource* | *7* | *0.78* | *0.607* | *7* | *0.38* | *0.911* |
| Habitat contrast x Round | 5 | 0.33 | 0.895 | 5 | 0.94 | 0.458 |
| Habitat contrast x PRE/POST | 1 | 0.16 | 0.684 | 1 | 0.34 | 0.557 |
| Habitat contrast x Resource | 1 | 0.02 | 0.890 | 1 | 1.42 | 0.230 |
| Round x Resource | 5 | 0.33 | 0.897 | 5 | 1.24 | 0.298 |
| PRE/POST x Resource | 1 | 0.32 | 0.571 | 1 | 0.71 | 0.411 |
| Residual | 114 |  |  | 115 |  |  |
| Roral | 188 |  |  | 189 |  |  |

**Table S5.** Results of permutational analysis of variance (Degrees of freedom *df*, pseudo-*F* and *P*-values) for the effects of the amount of SNG, habitat contrast and their interaction on rodent activity at trays with different resource types (crop seeds; weed seeds; beneficial prey; pest prey). Models were fitted for different resources separately.

|  |  | **Crop seeds** | | **Weed seeds** | | **Beneficial prey** | | **Pest prey** | |
| --- | --- | --- | --- | --- | --- | --- | --- | --- | --- |
|  | *df* | *F* | *P* | *F* | *P* | *F* | *P* | *F* | *P* |
| SNG cover | 1 | 0.72 | 0.420 | 2.31 | 0.195 | 0.25 | 0.633 | 1.77 | 0.239 |
| Habitat contrast | 1 | 0.28 | 0.877 | 0.12 | 0.748 | 0.02 | 0.886 | 1.62 | 0.259 |
| SNG cover x habitat contrast | 1 | 0.14 | 0.713 | 0.00 | 0.987 | 0.03 | 0.870 | 0.14 | 0.725 |
| *Landscape ID* | *7* | *0.52* | *0.792* | *0.84* | *0.603* | *0.53* | *0.788* | *1.34* | *0.397* |
| Residual | 5 |  |  |  |  |  |  |  |  |
| Total | 15 |  |  |  |  |  |  |  |  |

**Table S6.** Results of permutational analysis of variance (Degrees of freedom *df*, pseudo-*F* and *P*-values) for the effects of the amount of SNG, habitat contrast and their interaction on bird activity at trays with different resource types (crop seeds; weed seeds; beneficial prey; pest prey). Models were fitted for different resources separately.

|  |  | **Crop seeds** | | **Weed seeds** | | **Beneficial prey** | | **Pest prey** | |
| --- | --- | --- | --- | --- | --- | --- | --- | --- | --- |
|  | *df* | *F* | *P* | *F* | *P* | *F* | *P* | *F* | *P* |
| SNG cover | 1 | 0.83 | 0.401 | 1.25 | 0.323 | 0.04 | 0.855 | 0.01 | 0.937 |
| Habitat contrast | 1 | 2.47 | 0.173 | 3.07 | 0.143 | **5.57** | **0.045** | 1.29 | 0.294 |
| SNG cover x habitat contrast | 1 | 0.70 | 0.443 | 0.53 | 0.495 | 4.64 | 0.076 | 0.04 | 0.861 |
| *Landscape ID* | *7* | *1.20* | *0.375* | *1.37* | *0.312* | *1.44* | *0.303* | *1.05* | *0.495* |
| Residual | 5 |  |  |  |  |  |  |  |  |
| Total | 15 |  |  |  |  |  |  |  |  |

**Table S7.** Results of permutational analysis of variance (Degrees of freedom *df*, pseudo-*F* and *P*-values) for the effects of the amount of SNG, habitat contrast and their interactions on predation of different resource types (crop seeds; weed seeds; beneficial prey; pest prey) by rodents. Models were fitted for different resources separately.

|  |  | **Crop seeds** | | **Weed seeds** | | **Beneficial prey** | | **Pest prey** | |
| --- | --- | --- | --- | --- | --- | --- | --- | --- | --- |
|  | *df* | *F* | *P* | *F* | *P* | *F* | *P* | *F* | *P* |
| SNG cover | 1 | 0.09 | 0.777 | 3.84 | 0.112 | 0.48 | 0.517 | 2.01 | 0.223 |
| Habitat contrast | 1 | 2.10 | 0.202 | 0.56 | 0.488 | 0.04 | 0.847 | 1.91 | 0.219 |
| SNG cover x habitat contrast | 1 | 0.34 | 0.578 | 0.75 | 0.424 | 0.45 | 0.531 | 0.98 | 0.372 |
| *Landscape ID* | *7* | *0.21* | *0.962* | *1.22* | *0.427* | *2.37* | *0.194* | *3.20* | *0.107* |
| Residual | 5 |  |  |  |  |  |  |  |  |
| Total | 15 |  |  |  |  |  |  |  |  |

**Table S8.** Results of permutational analysis of variance (Degrees of freedom *df*, pseudo-*F* and *P*-values) for the effects of the amount of SNG, habitat contrast and their interactions on predation of different resource types (crop seeds; weed seeds; beneficial prey; pest prey) by birds. Models were fitted for different resources separately. Due to a low number of predation events no test could be performed for beneficial prey.

|  |  | **Crop seeds** | | **Weed seeds** | | **Beneficial prey** | | **Pest prey** | |
| --- | --- | --- | --- | --- | --- | --- | --- | --- | --- |
|  | *df* | *F* | *P* | *F* | *P* | *F* | *P* | *F* | *P* |
| SNG cover | 1 | 0.01 | 0.922 | 0.01 | 0.946 | - | - | 0.84 | 0.402 |
| Habitat contrast | 1 | 0.26 | 0.633 | **10.47** | **0.018** | - | - | 1.15 | 0.331 |
| SNG cover x habitat contrast | 1 | 0.31 | 0.603 | 6.31 | 0.057 | - | - | 0.45 | 0.539 |
| *Landscape ID* | *7* | *0.48* | *0.810* | *2.71* | *0.141* | *-* | *-* | *0.68* | *0.693* |
| Residual | 5 |  |  |  |  |  |  |  |  |
| Total | 15 |  |  |  |  |  |  |  |  |

**
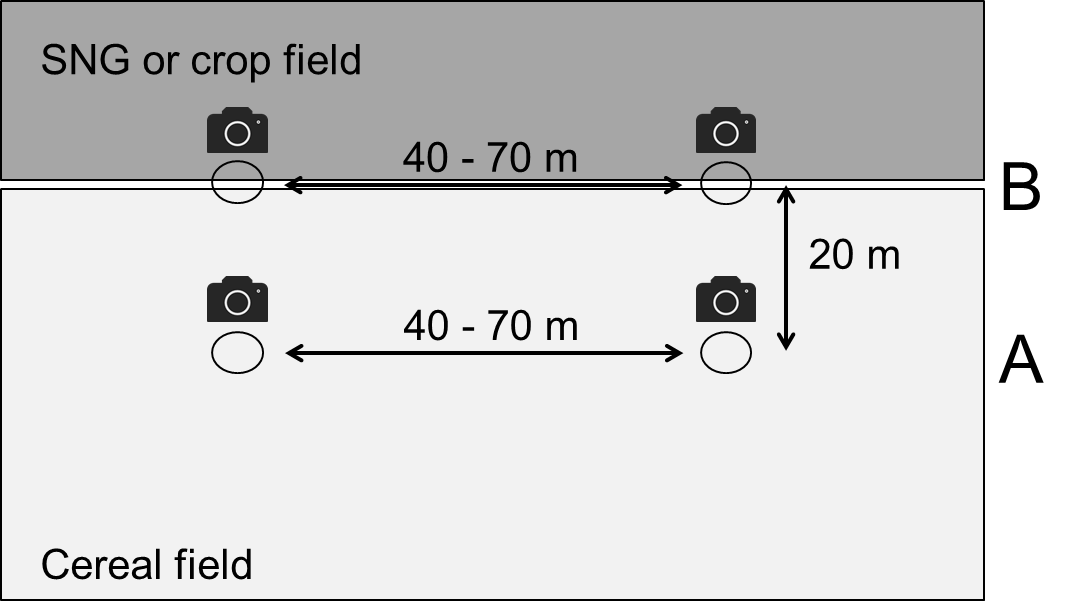
**

**Figure S1.** Placement of treatments within fields in A) spring and early summer (i.e. pre-harvest - round 1 to 3; Table S1) and B) in late summer and autumn (i.e. post-harvest – round 4 to 6; Table S1). Circles indicate flower pot trays and camera symbols represent motion-triggered wildlife cameras.


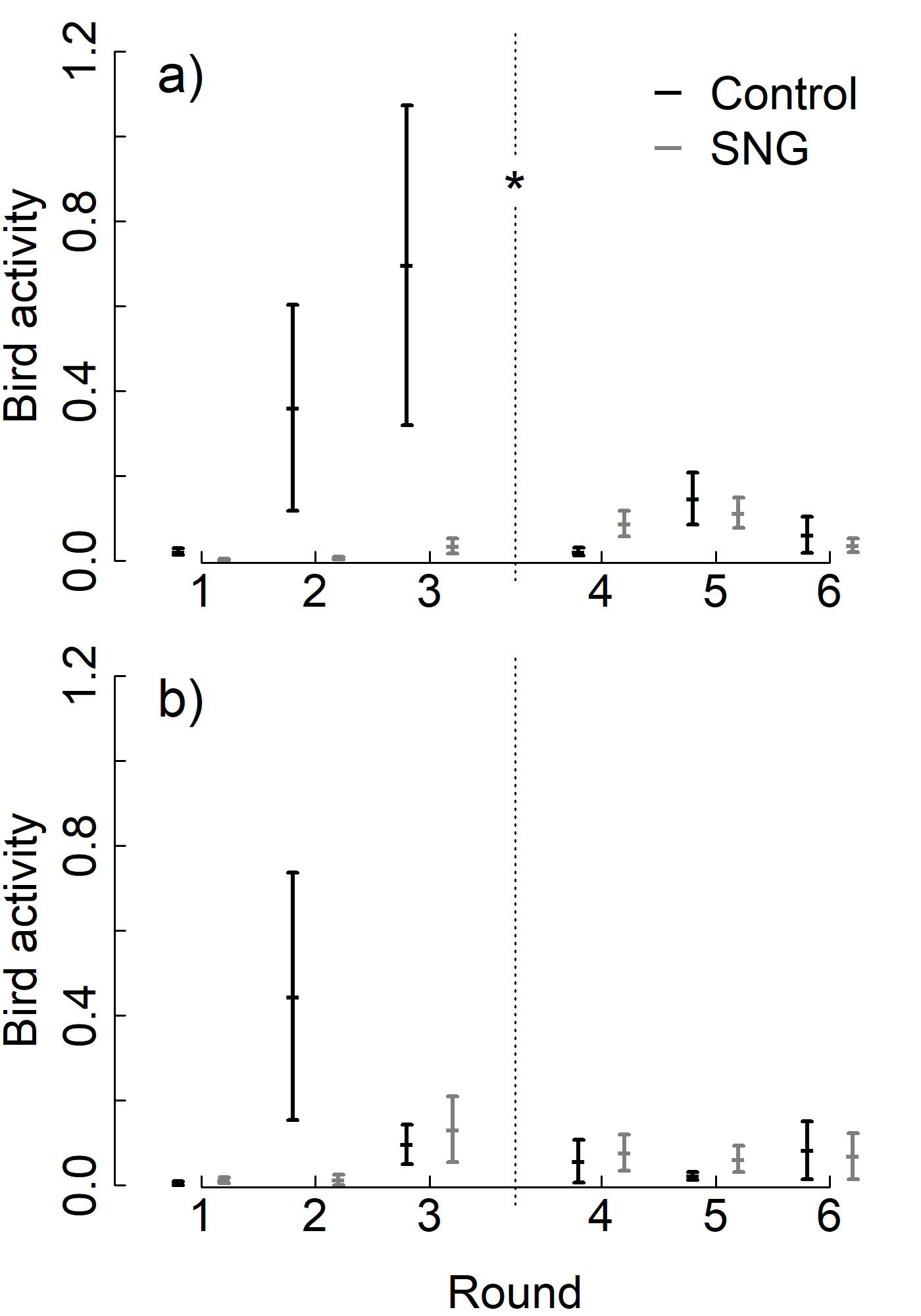


**Figure S2.** Bird activity at trays in SNG and control fields per sampling round. Mean (± 1SE) standardized bird activity (number of recorded pictures / hour) at trays with a) seeds and b) animal prey for fields adjacent to a semi-natural grassland (SNG; grey) and fields adjacent to another crop field (control; black) across the six rounds. The vertical line represents the separation between pre- (rounds 1 to 3) and post-harvest (rounds 4 to 6) and the asterisk indicates a significant effect (*P* ≤ 0.05) of the interaction of the pre- vs. post-harvest contrast with adjacent habitat type.
